# Consensus on the key characteristics of endocrine-disrupting chemicals as a basis for hazard identification

**DOI:** 10.1038/s41574-019-0273-8

**Published:** 2019-11-12

**Authors:** Michele A. La Merrill, Laura N. Vandenberg, Martyn T. Smith, William Goodson, Patience Browne, Heather B. Patisaul, Kathryn Z. Guyton, Andreas Kortenkamp, Vincent J. Cogliano, Tracey J. Woodruff, Linda Rieswijk, Hideko Sone, Kenneth S. Korach, Andrea C. Gore, Lauren Zeise, R. Thomas Zoeller

**Affiliations:** 1 Department of Environmental Toxicology, University of California, Davis, CA USA; 2 Department of Environmental Health Science, School of Public Health and Health Sciences, University of Masschusetts, Amherst, MA USA; 3 School of Public Health, University of California, Berkeley, CA USA; 4 California Pacific Medical Center Research Institute, Sutter Hospital, San Francisco, CA USA; 5 Environmental Directorate, Organisation for Economic Co-operation and Development, Paris, France; 6 Department of Biological Sciences, North Carolina State University, Raleigh, NC USA; 7 International Agency for Research on Cancer, World Health Organization, Lyon, France; 8 Department of Life Sciences, Brunel University, London, UK; 9 Office of the Science Advisor, United States Environmental Protection Agency, Washington, DC USA; 10 Program on Reproductive Health and the Environment, Department of Obstetrics, Gynecology and Reproductive Sciences, University of California, San Francisco, San Francisco, CA USA; 11 Institute of Data Science, Maastricht University, Maastricht, Netherlands; 12 Center for Health and Environmental Risk Research, National Institute for Environmental Studies, Ibaraki, Japan; 13 Receptor Biology, Section Reproductive and Developmental Biology Laboratory, National Institute of Environmental Health Science, Durham, NC USA; 14 Division of Pharmacology and Toxicology, University of Texas at Austin, Austin, TX USA; 15 Office of the Director, Office of Environmental Health Hazard Assessment of the California Environmental Protection Agency, Sacramento, CA USA; 16 Biology Department, University of Masschusetts, Amherst, MA USA

**Keywords:** Endocrinology, Risk factors, Chemical safety

## Abstract

Endocrine-disrupting chemicals (EDCs) are exogenous chemicals that interfere with hormone action, thereby increasing the risk of adverse health outcomes, including cancer, reproductive impairment, cognitive deficits and obesity. A complex literature of mechanistic studies provides evidence on the hazards of EDC exposure, yet there is no widely accepted systematic method to integrate these data to help identify EDC hazards. Inspired by work to improve hazard identification of carcinogens using key characteristics (KCs), we have developed ten KCs of EDCs based on our knowledge of hormone actions and EDC effects. In this Expert Consensus Statement, we describe the logic by which these KCs are identified and the assays that could be used to assess several of these KCs. We reflect on how these ten KCs can be used to identify, organize and utilize mechanistic data when evaluating chemicals as EDCs, and we use diethylstilbestrol, bisphenol A and perchlorate as examples to illustrate this approach.

## Abstract (web summary)

In this Expert Consensus Statement, the authors define 10 key characteristics (KCs) for endocrine-disrupting chemicals. They further describe the logic by which these KCs are identified and the assays that could be used to assess several of these KCs.

## Introduction

The endocrine system is composed of glands that secrete chemical messengers (hormones) that interact with specific targets (receptors). These interactions lead to the regulation of a vast set of functions, including growth, development, reproduction, energy balance, metabolism and body weight regulation^1^. Exogenous chemicals can inadvertently interfere with this complex communication system and cause adverse health effects. Throughout their lives, humans and other animals are exposed to a wide array of these so-called endocrine-disrupting chemicals (EDCs) through their encounters with work, consumer products, medications, natural resources, military service and other circumstances. This exposure can increase the risk of reproductive impairment^2–4^, cognitive deficits^5–7^, metabolic diseases and disorders^8,9^ and various cancers^10–13^, among others. The mechanisms by which hormones and EDCs exert specific actions are dependent on specific actions at the cellular and tissue levels as well as on circadian rhythms, seasonal changes, life stage and sex^14^. Moreover, the developmental, circadian or pulsatile pattern of hormone secretion can be an important component of their signalling mechanism and EDCs can interfere with this pattern^15–17^. Indeed, the risk of lifelong adverse health effects is enhanced when periods of EDC exposure coincide with the formation and differentiation of organ systems in early development^18^.

Although defining a chemical as an EDC is not a primary concern in all jurisdictions, an important key first step in governing exposures to chemicals with EDC properties is the identification of their intrinsic hazard. Regulatory agencies use various approaches to evaluate the available evidence, including for EDC identification^19–24^, but they can differ in the end points analysed and in their methods for gathering and interpreting the scientific evidence. Thus, standard, systematic approaches to organize and evaluate the often complex mechanistic data on a given chemical would reduce the likelihood of different jurisdictions arriving at different conclusions for hazard evaluations^25,26^.

Similar challenges were previously encountered in the evaluation of mechanistic data for cancer hazard identification. Therefore, the key characteristics (KCs) of human carcinogens were developed, providing a uniform basis for searching, organizing and evaluating mechanistic evidence to support the identification of carcinogens^27^. This KC-based approach is becoming widely applied by authoritative bodies, including the International Agency for Research on Cancer (IARC) and the National Toxicology Program, as according to the National Academies it “avoids a narrow focus on specific pathways and hypotheses and provides for a broad, holistic consideration of the mechanistic evidence”^28^. Indeed, the same National Academies report noted that KCs of other hazards, not just for carcinogens, should be developed^28^.

In this Expert Consensus Statement, we propose that chemicals that interfere with hormone action have identifiable KCs that relate to their ability to interact with key regulatory steps of hormone systems and that these KCs can be used to identify EDCs. Here, we identify the KCs that comprise the properties of all hormone systems. Ten KCs for EDCs are identified, representing the categories for the organization of the mechanistic evidence. Additionally, as with the KCs for carcinogens, the strength of the evidence is categorized for each KC during the hazard evaluation process^28^.

## Methods

We assembled an international group of experts with knowledge of hormone systems, EDCs, hazard evaluations and risk assessments, in vitro and in vivo screening tools, and carcinogenesis, with the goal of advancing the KC framework. A list of KCs for EDCs was developed by panel discussions to achieve consensus during a 2-day workshop and biweekly teleconferences. We next selected three chemicals to illustrate how these KCs can be used to identify the hazard of endocrine disruption: bisphenol A (BPA), diethylstilbestrol (DES) and perchlorate (Box 1). M.T.S., M.A.L.M. and R.T.Z. conceived the project and L.Z. facilitated the meeting funding. M.T.S. organized and chaired the workshop. M.A.L.M. developed the original set of KCs that were subsequently modified by the whole group. M.A.L.M. and R.T.Z. led the group discussions and subsequent biweekly teleconferences. All authors contributed ideas to these discussions and wrote specific sections of the manuscript.

Box 1 Sources of EDC exposures **Bisphenol A**

Bisphenol A was considered for use as a pharmaceutical synthetic oestrogen in the 1930s and is found today in a wide range of plastics, including medical and sports equipment, epoxy resins, the lining of food and beverage cans, dental sealants and other dental materials, paints, as a developer in thermal paper and in other papers, including food contact materials.

**Diethylstilbestrol**

Diethylstilbestrol was used to treat metastatic prostate cancer because of its oestrogenic effects in suppressing this hormone-responsive disease and was also prescribed to women during pregnancy to prevent miscarriage and premature labour, although it was ineffective for this purpose.

**Dichlorodiphenyltrichloroethane**

An organochlorine insecticide used to prevent diseases (such as malaria and typhus) carried by mosquito vectors that, as a result of widespread use, is a persistent organic pollutant found in many people worldwide.

**Dichlorodiphenyldichloroethylene**

A metabolite of dichlorodiphenyltrichloroethane that is also a persistent organic pollutant and common contaminant of the food supply, and is found in almost everyone worldwide.

**Di(2-ethylhexyl) phthalate**

A colourless and viscous plasticizer formerly used in the manufacture of polyvinyl chloride products, cosmetics, shower gels and shampoos.

**Methoxyacetic acid**

A metabolite of the solvent methoxyethanol that has been widely used in the semiconductor and painting industries.

**Perchlorate**

An inorganic ion that is widely manufactured for use in rocket propellant, matches, fireworks and other explosives, and is also a contaminant of hypochlorite bleach and drinking water supplies.

**Polychlorinated biphenyls**

A class of >200 chemicals formerly used in insulation and caulking that are persistent organic pollutants and common contaminants of the food supply.

EDC, endocrine-disupting chemical.

## Key characteristics of EDCs

**Fig. 1:**
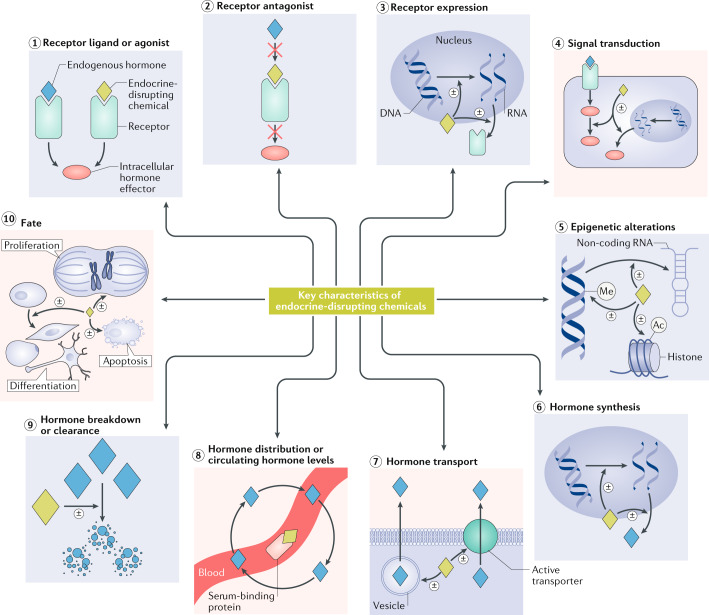
The key characteristics of endocrine-disrupting chemicals. Arrows identify the ten specific key characteristics (KCs) of endocrine-disrupting chemicals (EDCs). The ± symbol indicates that an EDC can increase or decrease processes and effects. KC1 states that an EDC can interact with or activate hormone receptors. KC2 states that an EDC can antagonize hormone receptors. KC3 states that an EDC can alter hormone receptor expression. KC4 states that an EDC can alter signal transduction (including changes in protein or RNA expression, post-translational modifications and/or ion flux) in hormone-responsive cells. KC5 states that an EDC can induce epigenetic modifications in hormone-producing or hormone-responsive cells. KC6 states that an EDC can alter hormone synthesis. KC7 states that an EDC can alter hormone transport across cell membranes. KC8 states that an EDC can alter hormone distribution or circulating hormone levels. KC9 states that an EDC can alter hormone metabolism or clearance. KC10 states that an EDC can alter the fate of hormone-producing or hormone-responsive cells. Depicted EDC actions include amplification and attenuation of effects. Ac, acetyl group; Me, methyl group.

The KCs of EDCs were developed by recognizing that there are common features of hormone regulation and action that are independent of the diversity of the effects of hormones during the life cycle. It follows that there are also features that characterize the actions of chemicals that interfere with hormone regulation and action. Thus, the ten KCs identified (Fig. 1; Table 1) are based on our evaluation of the scientific literature, both in the field of endocrinology and in endocrine disruptor research, including high-quality reports documenting the effects of chemical exposures on hormone systems (for example, see refs^29,30^). Indeed, as no internationally harmonized ‘list’ of EDCs exists, we could not simply use the strategy of identifying ‘commonalities’ among EDCs in terms of their mechanisms. Moreover, such a list of commonalities among chemicals would be biased toward chemicals that are well studied, such as carcinogens and reproductive or thyroid toxicants, which represent the bulk of EDC research. Therefore, we capitalized on the extensive knowledge of hormone action to generate the ten KCs described in this Expert Consensus Statement. This list of KCs reflects current scientific knowledge and will probably evolve over time with new scientific discovery.

**Table 1:** Key characteristics of EDCs and representative standardized tests that address them

Key characteristics	Examples of relevant streams of mechanistic evidence	Guideline description (species) [agency and guideline number]^a^
KC1. Interacts with or activates hormone receptors	Binding or agonism of hormone receptors	Androgen Receptor Binding (rat) [US EPA 890.1150]; Estrogen Receptor Binding (rat) [US EPA 890.1250, OECD TG 493]; Estrogen Receptor Transcriptional Activation (human stable transfection) [US EPA 890.1300, OECD TG 455]; Androgen Receptor Binding (rat) [US EPA 890.1150]; Androgen Receptor Transcriptional Activation (human stable transfection) [OECD TG 458]; Uterotrophic (rat) [US EPA 890.1600, OECD TG 440]; Hershberger [US EPA 890.1400, OECD TG 441]
KC2. Antagonizes hormone receptors	Antagonism of nuclear or cell surface hormone receptors	Estrogen Receptor Transcriptional Activation (human) [OECD TG 455]; Androgen Receptor Transcriptional Activation (human) [OECD TG 458]; Hershberger [US EPA 890.1400, OECD TG 441]
KC3. Alters hormone receptor expression	Abundance, distribution and degradation of hormone receptors	None
KC4. Alters signal transduction in hormone-responsive cells	Abundance of post-translational modifications, cofactors, transcription factors and transcripts, and activity of associated enzymes	None
KC5. Induces epigenetic modifications in hormone-producing or hormone- responsive cells	Chromatin modifications, DNA methylation and non-coding RNA expression	None
KC6. Alters hormone synthesis	Expression or activity of enzymes or substrates in hormone synthesis	Aromatase (human) [US EPA 890.1200]; Steroidogenesis (human) [US EPA 890.1550, OECD TG 456]
KC7. Alters hormone transport across cell membranes	Intracellular transport, vesicle dynamics or cellular secretion	None
KC8. Alters hormone distribution or circulating hormone levels	Blood protein expression and binding capacity, blood levels of pro-hormones and hormones	None
KC9. Alters hormone metabolism or clearance	Inactivation, breakdown, recycling, clearance, excretion or elimination of hormones	None
KC10. Alters fate of hormone-producing or hormone-responsive cells	Atrophy, hyperplasia, hypertrophy, differentiation, migration, proliferation or apoptosis	None

EDC, endocrine-disrupting chemical; OECD, Organisation for Economic Co-operation and Development; TG, test guideline; US EPA, US Environmental Protection Agency. ^a^Only assays that serve as the basis of regulatory decisions of the OECD and US EPA are provided.

### KC1: Interacts with or activates hormone receptors

All hormones act by binding to a specific receptor or receptors^1^. Inappropriate receptor activation can have profound negative effects on development and health, as illustrated by the formation of a scrotum and penis in genetic female humans exposed to androgens during gestation^31^. EDCs that inappropriately bind to and/or activate hormone receptors can produce adverse biological effects. There are numerous examples of chemicals that cause adverse effects after binding to nuclear hormone receptors. For example, EDCs that inappropriately activate the oestrogen receptors (ERα and ERβ) during development increase the risk of infertility in both sexes as well as reproductive tract cancer in women and prostate cancer in men^32^, in addition to other reproductive effects. Another example of an EDC that activates hormone receptors is that of dichlorodiphenyltrichloroethane (DDT; Box 1), which binds to ERα and ERβ^33^ and stimulates ER-dependent transcriptional activation and proliferation^34^ in a variety of species, including humans. Likewise, a specific hydroxylated congener of a polychlorinated biphenyl (PCB; Box 1) can activate human thyroid hormone receptor-β-mediated transcription^1,35^. EDCs can also activate cell membrane receptors of peptide and steroid hormones. For instance, DDT binds to the transmembrane domain of the follicle-stimulating hormone receptor, a G protein-coupled receptor (GPCR), to allosterically enhance its stimulation of cAMP production^36^.

### KC2: Antagonizes hormone receptors

EDCs can inhibit or block effects of endogenous hormones by acting as receptor antagonists^30^. Although antagonism of membrane hormone receptors or intracellular hormone receptors can occur (as exemplified by drug discovery efforts^37–39^), most exogenous chemical research into antagonization of receptors has focused on antagonization of nuclear hormone receptors. Nuclear receptors that act as ligand-dependent transcription factors by mediating genomic regulatory responses can be antagonized by some EDCs. For example, dichlorodiphenyldichloroethylene, an organochlorine pesticide (Box 1), inhibits androgen binding to the androgen receptor (AR) and inhibits androgen-dependent transactivation of the AR in human^40^ and rat prostrate cells^41^. Other organochlorine pesticides (such as lindane and dieldrin, which is closely related to the organochlorine insecticide aldrin) also inhibit dihydrotestosterone binding to the AR. As androgens are key regulators of male sexual differentiation during fetal development, disruption of androgen action through AR antagonism in this period can permanently demasculinize male fetuses and lead to malformations of the genital tract^42,43^.

### KC3: Alters hormone receptor expression

As hormone receptors mediate hormone actions^1^, their physiotemporal pattern of expression dictates their response to hormone signals^44,45^. For example, receptor abundance can determine both the concentration of hormones that produces an effect as well as the magnitude of the effect itself in some situations^46^. EDCs can modulate hormone receptor expression, internalization and degradation. For example, di(2‐ethylhexyl) phthalate decreases the expression of the mineralocorticoid (aldosteron) receptor (MR) in the testis of adult mice^47^, where under normal conditions, MR acts as a positive modulator of testosterone biosynthesis^48^. Further, BPA (Box 1) alters the expression of oestrogen, oxytocin and vasopressin receptors in brain nuclei^49–53^, and also reduces the proteasome-mediated degradation of ERβ^54^. The internalization of cell surface receptors is also disrupted by chemicals. For example, DDT prevents the internalization of the TSH receptor^55^.

### KC4: Alters signal transduction in hormone-responsive cells

The binding of a hormone to a receptor triggers specific intracellular responses that are dependent on the receptor and tissue-specific properties of the target cell. Signal transduction mediated through both membrane and intracellular hormone receptors is altered by some EDCs. The signalling of two classes of receptors will be discussed here as they are the most extensively studied in the field of endocrinology and have EDC effects; these receptors are cell surface membrane receptors (such as GPCRs, receptor kinases, and kinase-linked and ionotropic receptors) and nuclear steroid hormone receptors.

Ionotropic receptor signalling can be perturbed by EDCs. For example, BPA blocks low glucose-induced calcium signalling in isolated pancreatic glucagon-secreting α-cells from adult male mice^56^. Furthermore, in 2018 it was shown that chemicals in ultraviolet filters disrupt calcium signalling in human sperm^57,58^.

Some membrane GPCRs bind steroids; among these, G protein-coupled oestrogen receptor (GPER; previously called GPR30) signalling is the best studied regarding the EDC effects (for example, BPA^59^). Further, EDCs can attenuate or potentiate hormone action through signal transduction. For instance, in in vitro studies, the fungicide tolylfluanid impairs insulin action by reducing insulin receptor substrate 1 (ref.^60^), while methoxyacetic acid (Box 1) potentiates ligand-activated transcription and progesterone receptor-mediated transcription in a manner dependent on MEK1 and MEK2 activity^61^.

EDCs also affect signal transduction initiated by nuclear receptors. These effects include their interactions with coregulatory factors such as activators and repressors, which are a key part of the molecular machinery determining the downstream response to nuclear hormone receptor activation. The coregulatory factors for the steroid receptor coactivator (SRC) family are among the most studied in exogenous chemical research. For example, xenoestrogens (such as DES, PCBs, octylphenol and BPA; Box 1) induce the recruitment of SRC1 by ERα and ERβ in a dose-dependent manner^62^. BPA and its analogues also recruit SRC1 to thyroid hormone receptor-β^63^. Substantial evidence suggests that xenoestrogens, especially BPA, increase SRC1 expression, as shown in the rat hypothalamus^64,65^ and in human breast cancer cell lines^66^. Another EDC, 4-methylbenzylidene camphor (which is used in ultraviolet filters), also increases SRC1 expression in female rat hypothalamus^67^.

### KC5: Induces epigenetic modifications in hormone-producing or hormone-responsive cells

Hormones can exert permanent effects — especially during development and differentiation — by modifying epigenetic processes, including DNA and histone modifications and non-coding RNA expression. An EDC that interferes with hormone action can do so by interfering with the ability of a hormone to induce these epigenetic changes or by inducing these epigenetic changes to interfere with hormone action (such as by altering the expression or action of a hormone receptor or the transcription of hormone-responsive genes^68,69^). For example, the pesticide methoxychlor increases the expression of the DNA methyltransferase DNMT3B to hypermethylate DNA, including *ESR2* (which encodes ERβ) in the ovary of developmentally exposed rats^70^. In addition, di(2-ethylhexyl) phthalate inappropriately demethylates MR DNA in the testis of male mice^47^. EDCs can also change the expression of non-coding RNAs, as is seen with PCBs altering the developmental trajectories of hypothalamic microRNA expression in a sexually dimorphic manner^71^ as well as BPA and phthalates affecting microRNA expression in placental, Sertoli and breast cancer cell lines^72^.

Further to these data, a study on long non-coding RNAs found that oestradiol, BPA and DES induced HOX antisense intergenic RNA (termed HOTAIR) in human breast cancer cells^73^. In the presence of BPA and DES, the ER-binding region of the HOTAIR promoter was enriched by trimethylation on lysine 4 of histone 3 (H3K4) and by H3K4-specific methyltransferases, which are known to activate transcription^73^ (Table 2). Moreover, developmental BPA exposure enhances H3K4 trimethylation on genes, which are subsequently more sensitive to regulation by testosterone^68^. Oestradiol and DES also reduce trimethylation of lysine 27 on histone H3 in the developing uterus of rats^74^.

**Table 2:** Key characteristics of EDCs applied to three EDCs

Key characteristic	Mechanistic evidence for DES	Mechanistic evidence for BPA	Mechanistic evidence for perchlorate
KC1. Interacts with or activates hormone receptors	DES activates nuclear and membrane ER in mammals, including humans^56,109^	BPA activates nuclear ERs^140,141^, membrane ER^142^ and GPER^143^ in a variety of species	No evidence
KC2. Antagonizes hormone receptors	DES antagonizes oestrogen-related receptor-γ^109^	BPA antagonizes androgen receptor^144^	No evidence
KC3. Alters hormone receptor expression	Developmental DES exposure decreases ERβ expression in the developing female rat reproductive track^102^	BPA increases the expression of ER mRNA, and its location, in specific regions of the brain in mice exposed during gestation^145^	No evidence
KC4. Alters signal transduction in hormone-responsive cells	DES increases ERα-dependent transcriptional activity of enhancers proximal to a high density of ERα binding sites in the uteri of mice^7^; DES induces recruitment of SRC1 by ERα and ERβ in a dose-dependent manner^62^	BPA-induced proliferation of Sertoli TM4 cells is mediated by the induction of ERK phosphorylation; in a human testicular seminoma cell line (JKT-1), BPA activates cAMP-dependent and cGMP-dependent protein kinase pathways to phosphorylate CREB^59^	No evidence
KC5. Induces epigenetic modifications in hormone-producing or hormone-responsive cells	DES reduces trimethylation of H3K27 in the developing uteri of rats^74^; the ER-binding region of the long non-coding RNA HOTAIR promoter is enriched by trimethylation on H3K4 and by H3K4-specific methyltransferases in human breast cancer cells exposed to DES^73^	BPA affects promoter-specific methylation in brain, prostate and human breast cancer cells^73,146,147^; the ER-binding region of the long non-coding RNA HOTAIR promoter is enriched by trimethylation on H3K4 and by H3K4-specific methyltransferases in human breast cancer cells^73^; in mouse prostate, neonatal exposure to BPA activates the histone methyltransferase MLL1 to persistently increase H3K4 trimethylation at genes associated with prostate cancer^147^	No evidence
KC6. Alters hormone synthesis	DES decreases levels of the sex hormone precursor DHEA as well as downstream sex hormones, e.g. testosterone and oestrone in men^148^ and oestradiol in women^149^	BPA inhibits steroidogenesis in the rat testis^150^; BPA reduces cytochrome p450 aromatase levels and the expression of other steroidogenic regulatory proteins^151^	Perchlorate directly interferes with thyroid hormone synthesis by inhibiting iodide uptake through the human sodium–iodide symporter on thyrocytes, thereby reducing free intracellular iodine for the synthesis of thyroid hormone^152^
KC7. Alters hormone transport across cell membranes	No evidence	Low BPA doses reduce insulin secretion from vesicles of pancreatic islet β-cells^56^	No evidence
KC8. Alters hormone distribution or circulating levels of hormones	DES increases circulating levels of SHBG while decreasing circulating levels of LH, TSH, FSH, DHEA, testosterone and oestrone in humans^88,148,149,153^	In men, BPA exposure is associated with increased levels of SHBG^87,154^ as well as decreased circulating levels of androstenedione and free testosterone^87^	No evidence
KC9. Alters hormone metabolism or clearance	In utero exposure to DES correlates with a shift in women’s oestrogen metabolism with a relative decrease in 2-hydroxylation^155^	No evidence	No evidence
KC10. Alters fate of hormone-producing or hormone-responsive cells	Developmental exposure to DES increases proliferation in the developing female rat reproductive tract and abnormal mammary gland morphology^102,106^; DES induces keratinization of the vaginal epithelium of mice^104,105^	Developmental exposures to BPA alter the differentiation of mammary epithelial cells and increase the number of alveolar buds (structures that eventually produce milk in lactating females) in the mammary gland^156,157^; BPA also increases the proliferation index in the mammary gland^158^, pancreas^8^ and uterine endothelial cells^159^, among others	No evidence

BPA, bisphenol A; CREB, cAMP-response-element binding protein; DES, diethylstilbestrol; DHEA, dehydroepiandrosterone; EDC, endocrine-disrupting chemical; ER, oestrogen receptor; ERK, extracellular signal-regulated kinase; FSH, follicle-stimulating hormone; GPER, G protein-coupled oestrogen receptor; LH, luteinizing hormone; SHBG, sex hormone-binding globulin; SRC1, steroid receptor coactivator 1.

### KC6: Alters hormone synthesis

Hormone synthesis is regulated by both intracellular and distant endocrine feedback mechanisms. For example, thyroid hormone synthesis is controlled by a combination of TSH activation of their receptors on thyrotrophs as well as intracellular cAMP, iodine uptake and the activation of various enzymes^16^. After transcription and translation, proteins and peptide hormones are packaged into secretory vesicles where they can be stored^75^. By contrast, steroid hormone synthesis, including the transformation of a pro-hormone to a hormone, occurs more or less simultaneously with hormone activity^1^. Some EDCs are known to interfere with hormone synthesis. For example, perchlorate can block the uptake of iodine into thyroid cells, thereby inhibiting thyroid hormone synthesis^76^ (Box 1; Table 2) and phthalates can reduce testosterone synthesis in the fetal rat testis, resulting in testosterone insufficiency^77,78^.

By contrast, the herbicide atrazine apparently increases oestrogen synthesis in peripubertal male mice, as evidenced by a decrease in serum levels of testosterone coupled to an increase in circulating levels of oestrogen and in the activity of the aromatase that synthesizes oestrogen from testosterone^79^. Additionally, several neonicotinoid pesticides block the JAK–STAT3 pathway to enhance the promoter usage and expression of *CYP19* (aromatase), the gene that encodes the enzyme that converts testosterone to oestrogen^80^.

### KC7: Alters hormone transport across cell membranes

Due to their lipophilicity, steroid hormones (including oestrogens, androgens, progestins and adrenal steroids) can move through membranes passively. Other hormones (such as amine, peptide, protein and thyroid hormones) must be selectively transported across membranes either to gain entrance to and/or to exit the cell^81^. These selective and passive transport processes can be disrupted by EDCs. One well-delineated example of impaired selective transport by an exogenous chemical is low dose BPA, which reduces calcium entry into mouse pancreatic β-cells to reduce insulin secretion from vesicles^82^ (Table 2). Additionally, the anti-corrosive and fungicidal chemical imidazoline modulates ions to enhance insulin secretion^83^, and the passive secretion of corticosterone from rodent adrenal glands is reduced by low-dose dichlorodiphenyldichloroethylene^84^.

### KC8: Alters hormone distribution or circulating levels of hormones

Hormones typically circulate throughout the body in the blood at low concentrations, often in the parts per billion and trillion range^85^. Depending on its chemistry, a circulating hormone is either transported ‘free’ (not bound to serum protein) with or without conjugation (such as glucuronidation or sulfation) or is circulated bound to various proteins. EDCs can alter hormone bioavailability by interfering with the distribution of hormones in hormone-responsive tissues or with the circulation of hormones, including by displacing hormones from their serum binding proteins, which can lead to impaired active hormone delivery to target tissues. For example, BPA causes a concentration-dependent decrease in circulating levels of testosterone in male rats and men, and the pesticide malathion reduces testosterone levels in serum, testis and ovaries in several animals experimentally^30,86,87^. When DES is administered to men intravenously, reductions in total (a sixfold reduction) and free (a 20% reduction) testosterone and oestrogen (a fivefold reduction) are concurrent with an over sevenfold increase in serum concentration of sex hormone-binding globulin (SHBG)^88^ (Table 2).

### KC9: Alters hormone metabolism or clearance

The various hormone types (such as protein, peptide, steroid or thyroid) are inactivated differently. For example, after secretion, protein hormones (for example, gonadotropins) are inactivated when they are broken down by proteases in the blood. By contrast, steroid and thyroid hormones are metabolized by enzymes that render them hormonally inactive and increase their water solubility so that they can be removed from the bloodstream and excreted^1^.

EDCs can alter the rates of inactivation, including the metabolic degradation or clearance, of hormones, which could alter hormone concentrations and ultimately their activity. For example, a large number of chemicals activate glucuronidases, which increase thyroid hormone clearance from the blood^89^. Furthermore, sulfation by oestrogen sulfotransferase, which reduces the rate of oestrogen clearance from the blood, is inhibited by several coplanar hydroxylated PCB metabolites, a major metabolite of the fungicide hexachlorobenzene and several chlorophenolic wood preservatives^90^.

### KC10: Alters the fate of hormone-producing or hormone-responsive cells

Hormones affect tissue structure and organization by affecting cell fate (for example, cellular proliferation, migration or differentiation) and/or death (that is, apoptosis or necrosis) during development and adulthood^91–93^. In adulthood, many healthy endocrine organs have a fairly stable number of cells (including adrenals and pancreas), whereas other endocrine organs or hormone-responsive tissues depend on cell growth for normal function (such as the testicles to form sperm, the uterine endometrium and vaginal lining). EDCs can alter the total number or positioning of cells in hormone-producing or hormone-responsive tissues by disrupting or promoting differentiation, proliferation, migration or cell death. For example, thyroid hormone controls cell proliferation and apoptosis in the developing cerebellum and PCBs can interfere with thyroid hormone signalling to cause abnormal morphology later in life^94^. Female mice exposed to oxybenzone, a chemical ultraviolet filter found in personal care products, during pregnancy and lactation have increased mammary epithelial cell proliferation, which is observed even weeks after exposures cease^95^. In cultured human endometrial stromal cells, treatment with the antibacterial agent triclosan increases decidualization^96^. In the brain (anteroventral periventricular nucleus of the hypothalamus), developmental exposure to a PCB mixture substantially decreases the numbers of cells expressing ERα in adult female but not male rats^97^. Further, tebuconazole, a common fungicide, impairs placental migration, a process essential to placentation^98^.

## Application of the ten KCs

In the following sections we illustrate how the KCs can be used to organize and integrate mechanistic data with data on adverse endocrine effects in humans and in experimental models in an analysis designed to identify an EDC hazard. Sources of exposure, which could be considered to identify risk, are found in Box 1. Note that, for two of these examples, DES and BPA, substantial evidence exists for almost all the KCs yet for perchlorate strong evidence is available for only one KC (Table 2). Thus, the number of KCs associated with a specific exposure is not predictive of the EDC hazard as much as the relationship of the KC to hormone action.

### Example EDC: DES

In the human phenotype (of note, ‘human phenotype’ describes the clinical outcomes and epidemiology that characterize the adverse endocrine effects), women who were exposed in utero exhibit a higher incidence of breast cancer and clear cell carcinoma of the vagina^99^, in parallel to studies in mice (see below). Prenatal exposure to DES also is associated with disruption of the growth of hormone-sensitive structures in these daughters, with changes such as cellular abnormalities of the vaginal lining, increased breast size and abnormally shaped uteri in adolescents and young women^100,101^. Boys who were exposed to DES in utero are also prone to genital birth defects such as hypospadias and cryptorchidism^102^. Emerging evidence suggests that the effects of DES persist into the next (F2) generation; for example, the grandsons of women who took DES during pregnancy are at increased risk of genital defects^103^.

In the animal phenotype (of note, ‘animal phenotype’ describes the pathology and physiology observed in experimental animal models), DES was first identified as an oestrogen following the discovery that it induces keratinization of the vaginal epithelium — an oestrogenic effect — in mice^104,105^. In utero exposure to DES induces uterine deformities, reproductive tract cancer and abnormal mammary gland morphology in female rodents, while neonatal exposure leads to penile deformity and low sperm counts in male rodents^106^. DES increases body weight in livestock and subclinical doses induce obesity in rodents^107^. Multigenerational effects of DES have been documented in experimental animal studies, including vaginal adenocarcinomas observed in the granddaughters of mice that were exposed in adulthood^108^.

Mechanistic data (of note, the KCs are revealed in ‘mechanistic data’ from human and model cells, model organisms and through the use of biomarkers) show that DES exhibits nine of the ten KCs of an EDC and is noteworthy for the abundance of mechanistic data in humans (Table 2). DES is an agonist of nuclear and membrane ER^56,109^, including to SRC1 (ref.^62^) recruitment and epigenetic modifications^73,74^ that activate ER-responsive transcription. For example, DES induces gene expression of several oestrogen-responsive HOXA genes necessary for differentiation of human female reproductive tract cells, which is probably responsible for the metaplastic effects of DES^110,111^. Further, DES exposure alters circulating concentrations of hormone and SHBG in humans. When DES is administered to men intravenously, total and free levels of testosterone and oestrogen are reduced (a sixfold reduction, 20% reduction and fivefold reduction, respectively) concurrently with an over sevenfold increase in levels of SHBG^88,108,112^. The KCs of DES identified among mechanistic studies, along with the evidence from human and other animal studies, indicate that effects of DES on the reproductive axes of female and male humans and rodents are mediated by numerous DES KCs that perturb oestrogen action.

### Example EDC: BPA

In the human phenotype, there are now >100 epidemiology studies that show associations between BPA and adverse outcomes such as obesity, diabetes mellitus, female infertility, male sexual dysfunction, reduced birth weight and atypical neurobehaviours in children, among others^113^. Although many of these studies are cross-sectional, others are longitudinal, providing stronger evidence for causal relationships between exposures and effects.

In the animal phenotype, hundreds of studies demonstrate that, in rodents, even low doses of BPA can disrupt development of the brain, male and female reproductive tracts, and mammary gland and metabolic tissues under endocrine control, among others^114^. BPA can also induce precancerous and cancerous lesions of the mammary gland and prostate^115^.

Mechanistic data show that thousands of mechanistic scientific papers on BPA have been published that provide substantial evidence for nine of the ten KCs described above (Table 2). Experimental studies have shed light on the molecular mechanisms that explain the actions of BPA on human and animal phenotypes. These studies have revealed that BPA binds to ERα and ERβ, as well as GPER, membrane ER, thyroid hormone receptor and AR^116^. BPA then causes the enrichment of H3K4 trimethylation and H3K4-specific methyltransferases at the ER-binding region of the HOTAIR promoter, and these enrichments are known to activate transcription in oestrogen-responsive cells^73^. The activation of ER by BPA has multiple effects on organs in various species, for example, BPA activates ERβ-mediated ion flux, which underlies the reduction in insulin secretion from pancreatic β-cells in response to BPA treatment^82^. The aforementioned KCs of BPA are consistent with the known mechanisms that underlie the diverse adverse effects that have been associated with BPA exposure in humans and other animals.

### Example EDC: perchlorate

In the human phenotype, measures of urinary perchlorate — a biomarker of perchlorate exposure — in pregnant women are linked to reduced maternal levels of thyroid hormone in several, but not all, studies^117–120^. However, because newborn babies are uniquely sensitive to suppression of thyroid hormone synthesis^121^, the relationship between perchlorate exposure and circulating levels of thyroid hormones should be evaluated in this population. Among the five studies in which thyroid hormone levels were measured within a day of birth, there is consistent evidence that newborn babies from communities that have been exposed to perchlorate have lower T_4_ levels and higher TSH levels and thyroid disease than those from unexposed communities^122^. The sufficiency of dietary iodine intake in a population is also likely to be a contributing factor to the variability in epidemiological associations between circulating perchlorate levels and thyroid hormone levels.

In the animal phenotype, low doses of perchlorate reduces serum levels of T_4_ in pregnant rats and their pups^123^ and impairs synaptic function in the adult hippocampus^124^. This finding supports the negative association between perchlorate and levels of T_4_ in the human epidemiological studies described in the previous section.

Mechanistic data show that perchlorate has strong evidence for only one KC; yet, it is a critical KC as it provides biological plausibility to the human and animal findings. Perchlorate inhibits thyroid hormone synthesis (KC6; Table 2) by acting as a potent competitive inhibitor of iodide uptake through the sodium–iodide symporter from humans, rodents and other vertebrates^76,125^. This symporter protein normally transports iodide across cell membranes in the thyroid gland, gut lining, placenta, the lactating breast and the choroid plexus^126^. Collectively, the aforementioned research on perchlorate suggests that perchlorate reduces thyroid hormone levels in humans and other animals by limiting the amount of iodide available for the synthesis of these hormones.

## Application of the ten KCs to identify EDCs

The KCs of carcinogens have been successfully applied by the IARC Monographs Programme to evaluate the mechanistic data for >30 suspected carcinogens^127^. Within the context of IARC, carcinogens are identified by four separate data streams: human exposure, tumours in humans, tumours in animals and mechanistic. The mechanistic data identified through the KCs of carcinogens support the interpretation of these other data streams by freeing the reviewers from linking specific mechanisms to specific tumours, which is a nearly impossible task. Similarly, we envision that the KCs of EDCs will provide a structure for searching and organizing the relevant literature on mechanistic information in support of an evaluation of a chemical for endocrine disruption (Box 2). These KCs are not a checklist; any specific application of the KC approach to identifying EDCs will depend on the extent of evidence on the chemical as well as on the goals of the end-user (Box 3).

Depending on the end-users’ chosen parameters, such as the definition of the EDC used, the types and availability of data that can inform the evidence stream, and/or budget, end-users might wish to compress several KCs into a larger category or omit certain KCs in their EDC definition. It is critical in all circumstances to recognize that identifying an EDC is not merely counting the sum of KCs with supporting evidence. Hormones generally act through entire systems, and one KC might be sufficient to disrupt an entire system. Strong evidence for one KC could be enough to support identification of an EDC, as is illustrated by perchlorate, which only has one KC yet its endocrine-disrupting activity is strongly supported by human and experimental evidence. Hence, incorporation of mechanistic data into EDC hazard identification should not be thought of in terms of a minimum number of KC ‘hits’, but rather in terms of whether the chemical interferes with a key event in hormone action consistent with causing an adverse effect.

Box 2 Recommended uses of the key characteristics of EDCs

- Systematically search the scientific literature for mechanistic data by using appropriate combinations of keyword terms (such as controlled ontologies) to reproducibly identify end points relevant to the key characteristics (KCs).
- Screen the literature based on inclusion and exclusion criteria consistent with the KC definitions. The resulting included papers can then be further evaluated in more detail based on design and reporting features as defined by the end-user.
- Organize and integrate the gathered evidence on endocrine disruption across data streams. Such data might arise from molecular epidemiology studies, in vivo and in vitro tests in experimental models, high-throughput tests and in silico modelling. The latter data sources might be germane when the former mechanistic data sources are sparse.
- Characterize the mechanistic evidence for an endocrine-disrupting chemical (EDC) as ‘strong’, ‘limited’ or ‘inadequate’ to reflect the wide variance in the extent and quality of evidence for any given KC, and following the approach of the International Agency for Research on Cancer^139^.
- Free the investigator from ‘connecting the dots’ between the so-called ‘molecular initiating event’ and a specific mode of action or adverse outcome pathway. In combination with phenotypic data from epidemiological and animal studies, this strategy represents an important and practical addition to hazard identification.
- Evaluate the relevant literature for similar effects of disparate chemicals, enabling them to be grouped for possible classification as EDCs.

Box 3 Applications for key characteristics of EDCs **Identifying the hazards of a chemical as an endocrine-disrupting chemical (EDC)**

We recommend that the key characteristics (KCs) be used to support hazard identification, integrated with human epidemiological, clinical and animal data streams when available, for endocrine disruption.

**Grouping chemicals due to common hazard characteristics**

Identifying common distributions of KCs among chemicals might prove useful for integrating chemicals in cumulative hazard assessment, as is exemplified by the receptor activity-based toxic equivalency evaluation of a large class of structurally similar industrial chemicals known as dioxin-like chemicals. Toxic equivalency is a weighted system used by regulatory agencies to evaluate the hazard of mixtures of dioxin-like halogenated aromatic hydrocarbons, which bind the aryl hydrocarbon receptor with varying affinities and/or activities, where the most potent chemical, tetrachlorodibenzo-p-dioxin, is ranked 1. Historically, chemicals of concern have often been replaced by chemicals that are structurally similar and later found to pose similar risks. The KCs approach might help avoid a regrettable substitution by revealing undesirable mechanistic characteristics of potential replacement chemicals early in the product development phase.

**Providing a foundation for risk assessments**

The KCs enable objective consideration of the intrinsic hazard of EDCs, knowledge that can then be applied by individual geopolitical jurisdictions in the context of EDC exposure levels in their populations. Data mapped to a specific KC might support dose–response inferences for both data rich and data poor chemicals.

**Prioritizing data and testing protocol gaps**

Organizing mechanistic data into KCs will help elucidate those KCs where data are lacking. This will in turn help prioritize further studies of the chemical or chemicals of interest and the development of new testing protocols through expert-guided mapping of assays to KCs. As more and better assays are developed and validated, we envision the KCs being systematically evaluated through a series of specific tests.

## Assays to evaluate KCs

Some mechanistic assays have been developed to screen potential EDCs in a regulatory context, though these are limited to measuring chemicals that interact with sex steroid nuclear receptors or alter the synthesis of sex steroids (such as KCs 1, 2 and 6; Table 1). High-throughput mechanistic assays, on the other hand, are abundantly available in the suite of ToxCast^128^ and Tox21 (ref.^129^) assays, which screen thousands of chemicals for a variety of toxicity pathways, including endocrine disruption. These high-throughput assays have not undergone international validation, and therefore regulatory authorities use the resulting data only in certain contexts. For example, results of a ToxCast ER model that integrates data from 18 in vitro assays^130,131^ are accepted by the US Environmental Protection Agency in lieu of the rodent in vivo uterotrophic assays to screen for the oestrogenic effect of chemicals. In addition, high-throughput mechanistic data can be part of the data used to satisfy the European regulatory criteria of an endocrine disruptor^19^.

Despite the hundreds of toxicity end points in these high-throughput platforms, assays that assess several KCs of EDCs are not well represented or are absent. This absence of data presents an opportunity to use the KCs of EDCs to identify assay development needs. In addition to the high-throughput platforms used by federal agencies to screen for activity, the published in vitro, in vivo and chemoinformatics literature can inform the evaluation of EDC properties. Indeed, the KCs of EDCs can be used to identify search terms for the transparent acquisition of the extensive research emanating from academic and government research laboratories to contribute to the identification of EDCs (Box 2).

## Effect on risk assessment

Over the past several decades, innovative methods for identifying chemical interactions with a molecular target, such as a hormone receptor or enzyme, have become increasingly available. These interactions might initiate a sequence of downstream biological effects that lead to adverse outcomes, yet molecular effects and adverse responses are not usually evaluated in the same test. Establishing causal linkages between these molecular events and adverse outcomes therefore requires an organizational framework to evaluate biologically plausible connections between responses at different levels and from different methods. Mode of action analyses were developed in an attempt to link key events in a theoretical biological sequence (such as carcinogenicity and endocrine effects)^132,133^. Adverse outcome pathways are an expansion of mode of action concepts that include a molecular initiating event and an adverse outcome in an organism, which are linked by all key events measured at various levels of organization^134^. Both mode of action and adverse outcome pathways are linear, reductive models of complex physiology but might nonetheless be helpful for understanding how chemicals exert their toxic effects^135^.

A challenge to the practical application of mode of action and adverse outcome pathway approaches for chemical safety decision-making is the limitation in the current understanding of disease processes, which could be shown to be incorrect or incomplete^136^. This limitation was recognized by Sir Bradford Hill, who formalized the research of causality in humans while noting that “what is biologically plausible depends upon the biological knowledge of the day”^137^.

The KCs approach we describe herein can be viewed as identifying molecular initiating events or early key events in both mode of action and adverse outcome pathway frameworks based on our current knowledge of the molecular mechanisms of hormone action as well as the role of hormones in development, health and disease. Using KCs to assemble mechanistic data about a putative EDC does not require an exhaustive understanding of how the characteristics are causally linked to the endocrine response or an a priori hypothesis about the mode of action or adverse outcome pathways. Instead, the KCs are based on the common properties of hormone systems during vertebrate developmental and adult life stages. In this manner, the KC approach avoids “a narrow focus on specific pathways and hypotheses” and instead “provides for a broad, holistic consideration of the mechanistic evidence”^28^.

## Conclusions

The KCs of EDCs are the functional properties of agents that alter hormone action. This emphasis is both unique and powerful in that these KCs comprise the major mechanisms by which hormone systems can be disrupted, including by interfering with what they do, how they do it and how they are controlled. The literature on the fundamental and clinical actions of hormones is extremely large and the KCs, as we have proposed them, open the process of EDC hazard identification to this literature. An essential element of the KC approach is that it superimposes on the fundamental endocrine framework the mechanisms by which chemicals can interfere with these systems. The KC approach is also adaptable in that users can collapse KCs (such as combine KC1 and KC2) if their given situation is advanced by this. The ten KCs described herein can also be mapped to current and future assays used to identify EDCs.

The KCs are agnostic with respect to current or future knowledge of downstream health hazards and mechanistic pathways. As we learn more about chemical actions on endocrine systems, the KCs should be updated to reflect this new information. The value of this approach for EDCs, as for carcinogens, is that the inevitable mechanistic gaps in the delineation of the complete pathway from exposure to downstream health hazards need not hamper the identification of key chemical characteristics that lead to phenotypic end points. Even for the case of BPA, which has been more intensely studied than perhaps any other EDC^138^, there are gaps in our understanding of the complete molecular pathways by which BPA produces observed health effects. Indeed, the same can be said for well-known exposure–disease relationships such as cancers induced by tobacco smoking and developmental neurotoxicity from lead exposure. In the absence of ‘complete’ knowledge, the recommended KCs of EDCs approach can systematically identify gaps in data and therefore set research priorities through the process of hazard identification. The utility of this approach is evidenced by the 2018 paper demonstrating the effect of KCs in carcinogen hazard identification^127^.

We recommend that efforts to identify and classify a chemical as an EDC utilize our KCs of EDCs approach in conjunction with other data (including epidemiological and experimental animal data) as we exemplified in the section ‘Application of the ten KCs’. Similar to the KCs of carcinogens, the KCs of EDCs can distil complex EDC mechanistic research from human and animal studies into a transparent approach.

As highlighted by the demonstration that three different well-known EDCs exhibit different characteristics of interference with endocrine systems supporting 1–9 KCs, we emphasize that the KCs should not be used as a checklist. We identify applications for the KCs, including their use by agencies that have been charged with risk evaluation and EDC classification (Box 3). Future directions for the KCs of EDCs should include the development of a controlled ontology of search terms to facilitate their widespread application.

In conclusion, the KCs of EDCs approach provides a universal framework for organizing mechanistic evidence for hazard identification that can be the foundation for the implementation of EDC risk assessments worldwide. This approach is a highly novel advancement in the EDC field.

## Glossary

Risk: Probability that an agent will cause a disease or an adverse effect at a given level of exposure.
Differentiation: The process by which a multi-fate cell changes into a different more specialized cell.
Hazard: An agent that can cause disease or an adverse effect.
Carcinogens: Agents capable of causing cancer in living tissue.
Epigenetic processes: Changes to DNA caused by actions, such as acetylation and methylation of DNA and histones and expression of non-coding RNAs, which change gene availability and expression but do not change DNA sequence.
Distribution: The transfer of a substance from one location to another within the body following its absorption.
Clearance: The elimination and removal of a substance from a tissue or the organism as a whole.
Decidualization: A differentiation process that occurs in the uterus to promote placental formation.
Biomarkers: Measurable substances in an organism whose presence is indicative of some phenomenon such as disease, infection or environmental exposure.
ToxCast: A multi-year effort based at the US Environmental Protection Agency and launched in 2007 that uses automated chemical screening technologies, called high-throughput screening assays, to expose living cells, isolated proteins or other biological molecules to chemicals.
Tox21: A federal collaboration among the US Environmental Protection Agency, NIH (including National Center for Advancing Translational Sciences and the National Toxicology Program at the National Institute of Environmental Health Sciences) and the US Food and Drug Administration.
Mode of action: A functional or anatomical change, at the cellular level, resulting from the exposure of a living organism to a substance.
Adverse outcome pathways: The structured representation of biological events leading in a linear way to an adverse effect, beginning with a molecular initiating event and ending in an adverse outcome.
Molecular initiating event: The point at which a chemical first effects a biological target.
